# The development of a new corporate specific health risk measurement instrument, and its use in investigating the relationship between health and well-being and employee productivity

**DOI:** 10.1186/1476-069X-4-1

**Published:** 2005-01-28

**Authors:** Peter R Mills

**Affiliations:** 1 Vielife Ltd, 72–76 Borough High Street, London, SE1 1XF, UK; 2 Department of Respiratory Medicine, The Whittington Hospital, Highgate Hill, London, N19 5NF, UK

## Abstract

**Background:**

There is a growing body of evidence linking health and well-being to key business issues. Despite this, corporate uptake of workplace health promotion programmes has been slow outside the USA. One possible reason for this is the lack of a generally available health risk measure that is quick and easy to administer and produces data that is rich enough to inform and direct subsequent employee health promotional interventions.

**Methods:**

We report on the development and validation of the health and well-being (HWB) assessment, a free to use health risk appraisal questionnaire that has been specifically developed for use in the corporate setting. The HWB assessment focuses upon modifiable health issues that directly impact upon business drivers. Development involved interviews with business leaders to ascertain their key areas of focus, scientific and general literature review to find evidence for health status having an impact upon these areas, and end user testing.

Three UK-based organisations (insurance, telecommunications and consumer goods sectors) participated in the research. A total of 2224 employees completed the HWB assessment, the short-form 36 (SF-36) and the World Health Organisation Health and Work Performance questionnaire (WHO-HPQ) as part of the validation process.

**Results:**

The HWB assessment is a twenty item questionnaire covering ten areas of health and well-being. Completion of the HWB assessment generates a global health risk score and ten sub-scores corresponding to the ten areas covered. It is easy to use and quick to complete (average completion time was eight minutes) and showed good internal consistency and test-retest reliability. Statistically significant correlations with similar SF-36 variables were observed. A significant negative correlation between HWB score and productivity decrement, as measured by the WHO-HPQ, was observed (r = -0.4). Individuals with HWB scores above the 25^th ^percentile were more likely to achieve workplace productivity standards than those with scores below the 25^th ^percentile (OR 3.62, 95% confidence limits 2.93 – 4.47).

**Conclusion:**

The HWB assessment generates reliable business focused health risk data that can be used to direct and target appropriate interventions within corporate populations. It may also be useful in quantifying the financial impact health status issues have upon organisations.

## Background

The last decade has seen increasing interest in the health and well-being of the workforce. This has been driven partly by the increasing burden of direct healthcare costs, but also from a recognition that the economy within the developed world has appreciably changed[1,2]. The relative contribution of industry, compared with the service sector, to gross domestic product (GDP) has steadily declined since 1980. Industry now represents approximately 32% of GDP and services 66%[3]. With the shifting structure of the economy have come new challenges to occupational health physicians and human resource managers alike. A predominantly service-based economy has fewer tangible assets than its industrialised counterparts and the wealth that is generated is almost completely reliant upon the less tangible "human capital" of employees. It has therefore become an imperative to ensure that this human factor is optimised in order to meet business demands, especially during times of slow economic growth. In parallel with this greater business emphasis on the human factor has come a greater awareness of "post-industrialisation" health issues. These include stress and sleep dysfunction and conditions such as obesity and musculoskeletal pain that have arisen due to greater national wealth and an increasingly sedentary lifestyle [4-6].

The evidence for the impact of many lifestyle factors upon long-term health is overwhelming. Smoking, excess alcohol intake, poor nutritional status, a sedentary lifestyle and psychological distress have all been associated with numerous diseases [7-10]. Indeed it has been estimated that about a quarter of all healthcare costs can be attributed to conditions directly resulting from easily modifiable lifestyle factors[11]. As well as the long-term consequences of lifestyle on the genesis of disease, there is increasing evidence of the short-term effects such factors have upon individual performance and productivity. Smoking, high body mass index (BMI) and psychological distress have all been shown to have a major impact upon employee productivity at work [12-14]. Additionally, it has been shown that those individuals who are physically active in their leisure time are less likely to have short-term illness-related absence or experience musculoskeletal disorders [15-17].

With these issues gaining greater ascendancy in the corporate world, we saw a need for a short, easy to administer questionnaire that could capture this business critical health status information. By conducting a confidential survey of all employees, aggregated data can be used to provide a first step by which organisations can target and monitor appropriate population-based health interventions within their workforce. A key issue in conducting such surveys is maintaining individual privacy and ensuring confidentiality of information. The majority of US organisations who already conduct annual health surveys of their employee populations do so either via their occupational health departments or external third parties.

Although there are a number of general and specific health risk appraisal measures available for corporate use, they are either not well validated, suffer from being too long and cumbersome to administer, or cost an appreciable amount to use. In the case of health related quality of life measures, such as the SF-36, or specific stress indicators such as the general health questionnaire (GHQ), the data that is generated is not specific enough to direct health and well-being interventions within the corporate setting.

We report on the development and validation of the health and well-being (HWB) assessment, a free to use twenty item questionnaire. We also describe its use in assessing the impact employee health has upon productivity and performance.

## Methods

### Questionnaire development

Our principal aim was to develop a questionnaire that focused upon business pertinent health and well-being issues. A secondary aim was that it should be quick and easy to administer with the amalgamated results serving as a baseline from which employers can start to implement appropriate health promotion interventions within their employee populations.

We initially surveyed a sample of twelve business managers and executives to ascertain the key issues currently facing their organisations. Interviewees came from four different business sectors, namely (i) Technology (ii) Engineering (iii) Banking and Insurance and (iv) Public sector / Health. Interviews lasted no more than 30 minutes and were semi-structured, asking each interviewee to describe the key issues they faced in their day-to-day operations. We then searched the general and scientific literature for evidence of the effect health parameters have upon the issues identified. The key business issues facing our sample of corporate leaders could be categorised into four separate areas. Table 1 shows these four key areas and summarises how health and well-being can directly impact upon them. Searches of Medline, Embase and PsycINFO were made from 1990 onwards using "productivity", "customer satisfaction", "customer service", "absence", "absenteeism", "medical cost" and "business risk" as key words or phrases.

**Table 1:** Business Pertinent Health & Well-being Issues

Business Issue	**Increasing the productivity of the workforce**	**Improving customer service and satisfaction**	**Reducing the costs of ill-health**	**Reducing potential future business risks and liabilities**
Modifying effect of employee health and well-being on business issue	Many medical conditions and risks (e.g. diabetes, cardiovascular disease, migraine, pain, respiratory disease, high BMI, smoking, excess alcohol consumption) have a direct impact upon the day-to-day productivity of the workforce [12,14,19,22,33]. Sleep disturbance and disruption has a significant impact upon an individual's performance during the working day [34] Psychological distress / stress can have a profound impact upon worker productivity and performance [21,35,36].	Physical and mental health are component factors in developing employee commitment, job satisfaction and a "climate for service" within an organisation. Via these areas the health and well-being of employees is likely to be an indirect contributor to customer service and satisfaction [37-40]. Employee attitude and job satisfaction directly affect sales increases and customer satisfaction. [37].	High risk health status (e.g. poorly controlled medical conditions, sub-optimal nutritional status, lack of physical activity, high levels of psychological distress) are associated with greater medical care expenditure and higher levels of absence [13,28,41-44]. Musculoskeletal issues are the commonest cause of long-term sickness absence in manual workers. [45]. Corporate health and well-being programmes have been shown to produce a return on investment by decreasing medical care costs, worker compensation costs and absence [30,31,46-48].	Improving physical fitness within the workforce can reduce voluntary staff turnover [49]. Union backed employee stress-related liability claims have risen four-fold since 1999, posing a significant risk to the business [50]. Early retirement due to illness is placing a significant burden upon pension plans. Musculoskeletal and psychological issues are the two most frequent health related reasons for early retirement [51].

Domains within HWB assessment that help quantify issue	Medical Health Pain Body Mass Index Smoking Status Alcohol Consumption Sleep Status Symptoms of Stress	Overall HWB Score Symptoms of Stress Job Satisfaction	Medical Health Nutritional Balance Physical Activity Symptoms of Stress Pain Overall HWB Score	Physical Activity Symptoms of Stress Pain

Following interviews with executives and managers the key issues for businesses could generally be classified in one of four main areas; (i) increasing the productivity of the workforce, (ii) improving customer satisfaction, (iii) reducing the costs associated with employee ill-health and (iv) reducing potential future business risks and liabilities. For all four we found evidence for a modifying effect of health and well-being. The table shows the four identified business areas, the impact employee health and well-being has upon these areas and the domains included within the HWB that assess these areas.

Initial questionnaire development involved formulating items that corresponded with the health and well-being areas that impact upon the four key business issues. An initial set of 40 questions was tested and discussed in one to one interviews and focus group discussions. Thirty-five employees and managers, from companies in the four industry sectors described previously, participated in these sessions. Question changes and selection were an iterative process, with the final questionnaire formulated after three rounds of small group testing and one round of initial data collection from 100 volunteers. This background research and subsequent refinement led us to construct a 20-item questionnaire covering ten areas of health and well-being (see additional file 1: Appendix), which we termed sub-indices. The ten areas were:

• Medical health status

• The presence of pain

• Habitual levels of physical activity

• Nutritional balance

• Sleep status

• Symptoms of stress

• Job satisfaction

• Smoking status

• Alcohol consumption

• Body mass index

We used a combination of 5-point Likert scales and structured multi-choice questions. Six of the ten areas were assessed by single item "global" questions, including a modification of the non-exercise estimation of VO_2 _max question developed by Jackson and Ross[18]. Body mass index was scored according to desired ranges for the general population, as recommended by the World Health Organisation and the Department of Health. The remaining three areas (nutritional balance, sleep status and symptoms of stress) were assessed by multiple items. The number of possible responses to each of the sleep questions were reduced from an initial five responses to four, as the additional response was not found to be helpful as a discriminator. The checklist for the medical health question was developed according to current best available evidence for medical conditions impacting upon key business issues[12,19-23]. A single, non-scoring question on self perception of effectiveness at work was also included, not to replicate existing more detailed productivity measures, but to act as a global screening question to examine the relationship between health and work effectiveness in population analysis. The answer to each question was scored on a scale from zero to one-hundred. This was used as the relevant HWB sub-index for single item variables. The question scores for multi-item variables were averaged to give a zero to one-hundred sub-index score (see additional file 1: Appendix for full scoring algorithm). The overall HWB score was computed by summing and then averaging all ten sub-index scores, giving equal weight to each of the ten areas.

### Subjects

Three thousand full time employees of three UK-based organisations (one insurance company, one telecommunications company and one consumer goods manufacturer) were invited to complete the questionnaire via the internet. All data transmission utilised 128-bit encryption and all data storage was fully compliant with the UK Data Protection Act (1998). All participants were required to electronically sign an agreement for their anonymised data to be used in amalgamated format for purposes of research. A draw with a prize of a weekend break was offered as an incentive to participate for each company group. Thirty employees re-took the questionnaire four weeks after the initial completion date in order to provide test re-test data.

As well as completing the newly developed questionnaire, participants were also asked to concurrently complete the Short Form 36 (SF-36) and part B of the World Health Organisation's Health and Work Performance (WHO-HPQ) questionnaire in order to assess criterion validity[24,25]. The SF-36 was chosen as it is a "gold standard" health-related quality of life measure and because there is some overlap with the HWB assessment in the constructs it assesses. There are a number of well validated productivity measures available for use in the workplace, however the WHO-HPQ was chosen as it is a general productivity measure applicable to both those who have a diagnosed disease and those that do not [26]. Others have shown a clear relationship between health risk and productivity, it was therefore important for the validation of our questionnaire that this was replicated[12].

For each participant in the study details on age, gender, sickness absence in the preceding three months, company position, marital status and weekly working hours were also collected.

### Data analysis

All data analysis was carried out using *Statistica*, a statistical software package distributed by Statsoft Inc. (Tulsa, USA. )

## Results

Of the 3000 employees invited to participate in the study, 2224 completed the questionnaires (74% response rate). Online completion ensured that there were no missing data points in completed questionnaires. The mean age was 38.1 years (standard deviation 10.7). Fifty-nine per cent of respondents were female (see table 2). Age and gender of respondents accurately reflected the demographics of the three company populations as a whole. The average completion time for the HWB assessment was eight minutes.

**Table 2:** Participant characteristics

Gender	Male:	41%
	Female:	59%
Average Age (years)	38.1 (SD: 10.7)	

Marital Status	Single:	34%
	Married:	59%
	Separated / Widowed:	7%

Weekly Working Hours	<40:	47%
	40 – <50:	41%
	50 – <60:	9%
	60+:	3%

Annual Gross Income (£)	< 10,000:	13%
	10,000–19,999:	27%
	20,000–29,999:	30%
	30,000–49,999:	21%
	50,000+:	9%

Company Position	Junior:	49%
	Middle:	40%
	Senior:	11%

### Questionnaire validation

Principal components factor analysis of the three multi-item variables showed that for each the number of factors extracted was 1. Inter-item correlation, as assessed by the Cronbach α value, for each of these three scales was good (see table 3). General linear model analysis indicated that of age, gender, sickness absence, company position, marital status and weekly working hours the only variables that remained a significant predictor of HWB score were sickness absence and age (p < 0.0001 for both).

**Table 3:** α values for the multi-item variables of the HWB

**Scale**	**Number of items**	**Cronbach α**
Symptoms of stress	6	0.83
Sleep status	3	0.70
Nutritional balance	3	0.73

### Comparison with SF-36 scores

Significant correlations were seen between the SF-36 scales that assessed similar areas of health as the HWB sub-indices, namely, bodily pain vs. presence of pain (r = 0.79), mental health vs. symptoms of stress (r = 0.70) and mental component summary measure (MCS) vs. stress (r = 0.71). Additionally, there was a clear association between the overall HWB score and the General Health and Vitality scores of the SF-36 (r = 0.59 and 0.49 respectively). All SF-36 multi-item scales were significantly correlated with the overall HWB score (p ≤ 0.01).

### WHO-HPQ data

The 2224 individuals who completed the HWB assessment and the SF-36 also completed part B of the WHO-HPQ. The output from the WHO-HPQ is a calculated productivity decrement for each respondent, i.e. the proportion of the week that the individual is not working optimally, either because they are absent or because they are not working effectively (so called "presenteeism")[24]. Mean productivity decrement for the population was 26.4% of weekly working time (SD 20.9), median 20% (25^th ^percentile was 10% and 75^th ^percentile was 33.5%)

A negative correlation between the HWB score and calculated productivity decrement was observed (r = -0.4, p < 0.0001), i.e. better health status, as measured by the HWB assessment, was associated with less weekly productivity decrement.

General linear model analysis indicated that age and the overall HWB score were the only two variables that remained as significant predictors of weekly productivity decrement (p < 0.0001 for both).

The 75^th ^percentile figure of 33.5% productivity decrement per week was taken as the cut-off for achieving the productivity standard within the current population.

Similarly, the lower quartile HWB score of 52.1 was used as the cut-off to define poor health. 2 × 2 table analysis using these cut-offs demonstrates an odds ratio of 3.62 (95% confidence limits, 2.93 to 4.47) for making the productivity standard if HWB score is above the lower quartile value, Chi squares 158.82 (Yates Corrected), p < 0.0001.

There was a significant correlation between the single question on effectiveness contained in the HWB assessment and the productivity decrement, as calculated by the WHO-HPQ, (r = -0.59, p < 0.0001).

### Test re-test validity for the HWB assessment

Thirty individuals re-took the HWB assessment four weeks after their original completion date. During this time no information or intervention with regard to health and well-being was delivered to them. The correlation between HWB scores at both time points was excellent (r = 0.90), with no significant differences between mean scores or variance of the data sets.

### HWB scores across the population

Table 4 gives the means, medians, standard deviations and inter-quartile values for the HWB score and sub-indices. The distribution of the HWB score was normal, therefore parametric measures were used to analyse differences between independent groups (t-test). There were no significant differences between the HWB score of males and females or between those who typically worked more than 40 hours and those who did not. There was, however, a significant difference in HWB score between those in senior positions within the company and those within junior positions (mean HWB scores 62.9 and 60.7 respectively, p < 0.001). Similarly, those who had less than three days sickness absence in the preceding three months had better HWB scores than those who had more sickness absence (means scores 64.0 and 55.2 respectively, p < 0.0001).

**Table 4:** Overall HWB score plus the ten component sub-index scores for the 2224 questionnaire respondents.

	Mean score	Median score	Standard deviation	25^th ^percentile	75^th ^percentile
HWB score	61.4	62.1	13.7	52.1	71.0
Medical health	62.4	100	41.3	25.0	100
Pain	71.2	75.0	21.9	50.0	75.0
Physical activity	26.3	0	38.1	0	50.0
Nutrition	57.5	58.3	19.0	41.7	75.0
Sleep	62.3	66.7	23.8	50.0	83.3
Stress	55.7	58.3	18.2	41.7	70.8
Job satisfaction	59.0	75.0	30.2	50.0	75.0
Smoking status	77.5	100	41.8	100	100
Alcohol consumption	92.2	100	26.8	100	100
Body Mass Index score	49.7	25.0	42.2	25.0	100

## Discussion

The association between employee health status and costs incurred by employers is incontrovertible. Numerous studies have clearly shown how health risk factors directly impact upon medical care costs, short- and long-term absence and workers' compensation[11,27-29]. Additionally, more recent research is confirming what many of us "intuitively" knew; that the health and well-being of the workforce has a direct impact upon work performance[12]. Despite this growing body of evidence, many corporations have been slow to implement appropriate measures to assess, intervene and improve the health of their workforce. The reason for this inertia is unclear, especially as corporate health promotion and management programmes have repeatedly been shown to generate a return on investment (ROI) [30-32]. A possible explanation may be that whilst medical care costs are inexorably increasing, by focusing solely upon costs and cost savings we miss capturing corporate leaders' imagination and vision. Combining the message of cost savings with productivity and performance enhancements may just strike the right balance. Measures such as the WHO-HPQ now allow us to objectively measure productivity and, as we have confirmed in this paper, health risk status is an integral component of this construct.

As already mentioned, although well-established questionnaires have been extensively validated in many different populations the data that is generated is often of limited value in specifically directing health and well-being interventions. We have presented the first steps of the development and validation of a health risk appraisal measure that has been specifically designed for use in the corporate setting. As well as having good content, criterion and construct validity, the generated data can help health promotion specialists develop appropriate and targeted interventions for the respondent population. The questionnaire provides information on areas such as nutritional choices, levels of habitual physical activity, sleep difficulties and stress symptoms. Amalgamated answers can be used to ensure the correct and most appropriate health interventions are delivered to the population being assessed. In addition, the single question on work effectiveness can be used to confirm the link between the health of the population being studied and their performance, prior to more in-depth evaluations of productivity such as can be made with a specific productivity measure.

One would naturally expect those individuals who have taken more time off due to illness to have worse health than those who have been absent for less time. We have demonstrated that sickness absence in the preceding three months is a significant predictor of HWB score and remains so when other variables are controlled for. This is an indication that the HWB assessment is indeed measuring the health and well-being issues that are critical to businesses as a whole. Further confirmation of the discriminant validity of the HWB assessment is needed, however a suggestion that it can detect real differences in health status between groups is also seen in the significantly better scores observed between those with more senior positions as compared with those in junior positions. This difference possibly reflects the better financial rewards, the better access to healthy alternatives and the superior levels of job control associated with more senior corporate positions.

The fact that the HWB score and sub-indices were significantly correlated with the broadly similar SF-36 multi-tem scales is an indication that the majority of the constructs assessed by the SF-36 are at least partially reflected in the HWB.

Productivity whilst at work can be influenced by a multitude of different factors, however as demonstrated by Burton and colleagues, health is a major contributor[12]. Our study has confirmed this clear relationship between level of health risk and productivity decrement, which remains significant even when other possible confounders are taken into account. Additionally, we have demonstrated that there is an odds ratio of 3.62 of making the productivity standard for those with good health as compared with those with poor health. This information can quite easily be used by corporations to model future productivity gains and to calculate a likely ROI for the institution of a health promotion programme.

Although these initial results appear promising, data collection from a larger employee sample, from different sectors and incorporating a wider age range, is necessary in order to confirm that our observations still hold true. Normalising the scoring (as is often performed with SF-36 data) would also make interpretation easier and more user friendly. Additionally, longitudinal data on whether the HWB assessment can be used as a predictive tool for populations, and hence provide businesses with visibility on how their employee health status issues are likely to affect their bottom line, is the logical next step. This process is already underway in four multinational organisations with populations in both the USA and the UK and is being overseen by the Institute for Health and Productivity Management (IHPM).

## Conclusion

In summary we present a new health risk measure, the Health and Well-being Assessment (HWB), which has the following key features:

(i) has been specifically designed for the corporate environment addressing the health and well-being issues that affect key business drivers

(ii) is quick, easy and free to use

(iii) the generated data is useful for guiding future interventions

By combining medical health issues with other more "lifestyle" and well-being focused areas within a short, easy to use questionnaire we believe that we have created a useful corporate tool.

## List of Abbreviations

GHQ – General Health Questionnaire

HWB – Health and well-being

ROI – Return on Investment

SF-36 – Short Form 36 questionnaire

WHO-HPQ – World Health Organisation Health and Work Productivity Questionnaire

## Competing Interests

PM has a part-time salaried role with health and well-being business consultants Vielife.

No other financial competing interests

No non-financial competing interests.

## Authors' contributions

PM performed all of the work that is contained within this paper.

## Supplementary Material

Additional File 1 Health & Well-being Questionnaire Questionnaire and scoring algorithms. Click here for file
