# Supplementary material for: The development of a new corporate specific health risk measurement instrument, and its use in investigating the relationship between health and well-being and employee productivity
Source: Environ Health. 2005 Jan 28;4:1. doi: 10.1186/1476-069X-4-1 (PMC548523; doi:10.1186/1476-069X-4-1)
Supplement: Additional File 1 — Health & Well-being Questionnaire Questionnaire and scoring algorithms. [file 1476-069X-4-1-S1.doc]

**Appendix:**

***Health & Well-being Questionnaire***

The following questionnaire was completed online by all study participants. Each question had explanatory text associated with it that gave reasons for asking the question and appropriate examples to aid understanding. The numbers in square brackets represent the “score” attributed to the possible responses to each question (full scoring algorithm given at end of document).

# Q1

Background details

Male □ Female □

Height________ Weight________

# Q2

Do you have, or are you being treated for, any of the following conditions?

Please tick all that apply

□ Anxiety

□ Arthritis

□ Asthma, bronchitis or emphysema

□ Back or spinal problems

□ Cancer

□ Depression or bipolar disorder

□ Diabetes

□ Heart disease

□ High blood pressure

□ High cholesterol

□ Migraine Headaches

□ Sinusitis or allergic rhinitis (hayfever)

□ Any other serious health problem for which you are receiving medical treatment

# Q3

On average how many units of alcohol do you consume per week

□ I do not drink alcohol [100]

□ 0 to 7 [100]

□ 8 to 14 [100]

□ 15 to 20 [100 if male] [0 if female]

□ 21 or more [0]

**Q4**

Do you smoke every day

□ No [100]

□ Yes [0]

# Q5

How much bodily pain have you experienced during the last 3 months?

□ None [100]

□ Mild [75]

□ Moderate [50]

□ Severe [25]

□ Very Severe [0]

# Q6

Which of the following five statements best describes your usual level of physical activity?

□ I avoid exerting myself whenever possible. I use the lift / elevator rather than taking the stairs and drive rather than walk. [0]

□ I often walk places and occasionally exercise enough to cause myself to breathe more heavily than usual, but do this for less than 30 minutes per day [0]

□ I take regular moderate intensity activity (such as cycling, brisk walking, playing golf or gardening) that causes me to breathe more heavily than usual and sweat. On average I do this for 30 minutes a day on most days of the week [50]

□ I regularly do high intensity physical activity, such as running, swimming lengths or gym work. I do this for between 30 and 60 minutes a week [75]

□ I regularly do high intensity physical activity, such as running, swimming lengths or gym work. I do this for more than an hour a week [100]

# Q7

How many portions of fibre do you eat a day?

□ 1 or none [0]

□ 2 or 3 [25]

□ 3 or 4 [50]

□ 5 [75]

□ 6 or more [100]

# Q8

How often do you eat a portion of fruit or vegetables?

□ Rarely or never [0]

□ Occasionally, less than once per day [25]

□ 1 to 2 times per day [50]

□ 3 to 4 times per day [75]

□ 5 or more times a day [100]

# Q9

When choosing foods for your meal, do you usually select high-fat or low-fat foods?

□ I choose high-fat foods nearly all the time [0]

□ I choose high-fat foods most of the time [25]

□ I choose both high- and low-fat foods equally as often [50]

□ I choose low-fat foods most of the time [75]

□ I choose low fat foods all of the time [100]

# Q10

On a scale of 1 through to 5 how satisfied are you with your current job?

1 = Not very satisfied

2 = A little satisfied

3 = Moderately satisfied

4 = Satisfied

5 = Very satisfied

1 2 3 4 5

□ □ □ □ □

[0] [25] [50] [75] [100]

Please rate the following four statements on the 1 through to 5 scale, where

1 = Not at all

2 = A little

3 = A moderately amount

4 = Most of the time

5 = All of the time

**Q11**

How much of the time during the last 3 months have you felt calm and

peaceful?

1 2 3 4 5

□ □ □ □ □

[0] [25] [50] [75] [100]

# Q12

How much of the time during the last 3 months did you have a lot of energy?

1 2 3 4 5

□ □ □ □ □

[0] [25] [50] [75] [100]

# Q13

How much of the time during the last 3 months have you felt depressed or sad?

1 2 3 4 5

□ □ □ □ □

[100] [75] [50] [25] [0]

# Q14

How much of the time during the last 3 months have you felt happy?

1 2 3 4 5

□ □ □ □ □

[0] [25] [50] [75] [100]

# Q15

How do you feel about the coming six months?

□ Very concerned and worried, the coming six months are going to be very difficult for me and I’m not sure how well I’ll cope [0]

□ Moderately concerned and worried, the coming six months are going to be difficult, but I’m sure I’ll cope [25]

□ Neither concerned nor optimistic, the coming six months are going to be pretty much the same as usual for me [50]

□ Moderately optimistic, I think the coming six months are going to be good for me [75]

□ Very optimistic. I am looking forward to the coming six months, everything is going right for me [100]

# Q16

During the last 3 months how much of the time have you felt overwhelmed with pressure or stress from responsibilities, circumstances or relationships?

1 = Not at all

2 = A little of the time

3 = A moderate amount of the time

4 = Most of the time

5 = All of the time

1 2 3 4 5

□ □ □ □ □

[100] [75] [50] [25] [0]

# Q17

On average how many hours of sleep do you get a night?

□ 5 or less hours [0]

□ More than 5 hours but less than 7 hours [50]

□ 7 to 8 hours [100]

□ More than 8 hours [100]

# Q18

In general how happy are you with the amount and quality of sleep that you get?

□ Very happy, I sleep well [100]

□ Mostly happy, I usually sleep well but occasionally I have difficulties [75]

□ A little unhappy, I often have sleep difficulties [25]

□ Very unhappy, I regularly have sleep difficulties and usually sleep very poorly [0]

# Q19

How refreshed and restored do you feel ½ an hour after getting up in the morning?

□ Completely refreshed and restored [100]

□ A little tired but generally refreshed [75]

□ Rather un-refreshed, but able to function [25]

□ Completely exhausted and un-refreshed. [0]

Consider your work responsibilities and how effective you are in accomplishing them. Please answer the following question on the 1 though to 5 scale.

**Q20**

How effective in your work have you been over the last 3 months?

1 = Not effective

2 = A little effective

3 = Moderately effective

4 = Quite effective

5 = Highly effective

1 2 3 4 5

□ □ □ □ □

[0] [25] [50] [75] [100]

The following additional background / demographic information was collected either from the individual or from the human resources department:

1. Date of birth
2. Number of sickness absence days in the last 6 months

**Scoring of questionnaire:**

**Medical Health Status:**

| Number of medical conditions | Score |
| --- | --- |
| 0 | 100 |
| 1 | 75 |
| 2 | 50 |
| 3 | 25 |
| 4+ | 0 |

**Bodily Pain**

Scored according to answer given to Q5

**Physical Activity**

Scored according to answer given to Q6

**Nutrition**

Sum of scores from Qs 7, 8 and 9 divided by 3

**Sleep**

Sum of scores from Qs 17, 18 and 19 divided by 3

**Stress**

Sum of scores from Qs 11, 12,13,14,15 and 16 divided by 6

**Job Satisfaction**

Scored according to answer to Q10

**Smoking**

Scored according to answer to Q4

**Alcohol**

Scored according to answer to Q3

**Body mass index**

| Body Mass Index | Score |
| --- | --- |
| <18.5 | 50 |
| 18.5 to <25 | 100 |
| 25 to <30 | 25 |
| ≥ 30 | 0 |

This questionnaire and its associated scoring is the copyright of Vielife Ltd. It is free to use, however all we ask is that you inform us of where you intend to use it and share any pertinent research findings (contact information: email: [p.mills@vielife.com](mailto:p.mills@vielife.com) telephone: +44-20-7571 3836).
